# The Evolution of Symbiosis in *Staphylococcus epidermidis*: From a Protective Mutualist to a Parasitic Pathogen

**DOI:** 10.3390/biom16020334

**Published:** 2026-02-23

**Authors:** Stefanie Au, William Dela Cruz, Mehzabin Lala, Srinivasan Karthikeyan, Vishwanath Venketaraman

**Affiliations:** Department of Microbiology/Immunology, College of Osteopathic Medicine of the Pacific, Western University of Health Sciences, 309 E. Second St., Pomona, CA 91766-1854, USA; stefanie.au@westernu.edu (S.A.); william.delacruz@westernu.edu (W.D.C.); seenu0610@gmail.com (S.K.)

**Keywords:** Staphyloccocus, *S. epidermidis*, commensal bacteria, *S. aureus*, symbiotic bacteria, multidrug-resistant *S. epidermidis*, MDRSE

## Abstract

*Staphylococcus epidermidis* is more often known as a human skin commensal, serving as a primary protective bacterium on the skin’s surface. However, more recent literature highlights the role of *S. epidermidis* as a nosocomial pathogen and a multidrug-resistant organism that poses a global threat. The evolution of *S. epidermidis* can be owed to its accumulation of resistance mechanisms, including adhesion, biofilm formation, genomic islands, phage elements, integrated plasmids, and quorum sensing. It is suspected that through gene transfer, *S. epidermidis* is partially responsible for the feared multidrug-resistant *Staphylococcus aureus* through the *mecA* gene and many other genomic island transfers. Overall, prolonged nosocomial exposure and misuse of antibiotics have driven dramatic genomic remodeling in *S. epidermidis*, characterized by many methods of genetic recombination, *SCCmec* and insertion sequence acquisition, and accumulation of multiple resistance genes. Our review reviews the role of *S. epidermidis* as both a commensal and a pathogenic bacterium, summarizes the genes responsible for its multidrug resistance, and describes methods of combatting its invasion.

## 1. Introduction

*Staphylococcus epidermidis* (*S. epidermidis*) is a coagulase-negative member of the *Staphylococcus* genus. Historically, *S. epidermidis* has been known as a harmless mucus membrane commensal, serving as the primary protective bacterium on the skin surface. However, more recent literature highlights the role of *S. epidermidis* as a nosocomial pathogen. This species is capable of causing many fatal diseases, such as bloodstream infections that may lead to infective endocarditis and possible sepsis. While these diseases may primarily affect immunocompromised patients or patients with implanted prosthetic devices, their ability to evolve into a multidrug-resistant organism poses a global threat to the immunocompetent as well.

Furthermore, *S. epidermidis* has many methods of diversifying its virulence factors, and thus, the species is constantly evolving. This literature review will investigate the genetic factors contributing to the evolution of *S. epidermidis*, with particular emphasis on the similarities to *S. aureus*, biofilm formation, and antibiotic resistance, highlighting the role in its pathogenicity. Understanding these factors is pivotal to figuring out how a protective, commensal bacterium can evolve into a clinically significant pathogen.

## 2. Background

### 2.1. Commensal S. epidermidis

To humans’ benefit, *S. epidermidis* has the capability of promoting skin barrier integrity, modulating innate and adaptive immunity, and preventing skin flora colonization by harmful pathogens. *S. epidermidis* has appeared to be beneficial to its host by secreting bacteriocins, antimicrobial peptides that prevent colonization of other harmful bacteria [1]. A study conducted in mice demonstrated that *S. epidermidis* induces IL-17A+ CD8+ T cells in the absence of infection that have the potential to enhance innate barrier immunity and limit pathogen invasion [2]. These typically inflammatory cytokines are believed to have led to a homeostatic mechanism in which dendritic cells can recognize when the skin barrier is compromised.

Moreover, *S. epidermidis* secretes a sphingomyelinase, which assists the bacteria in producing ceramides, which provide the human skin with hydration and are a major ingredient of dermatology products. It is also believed that sphingomyelinase produces nutrients for *S. epidermidis* bacteria to thrive and prevents the skin from being colonized by bacteria such as MRSA [3]. Similarly, *S. epidermidis* produces butyrate, a fatty acid metabolite shown to protect the epidermis from UVB-induced IL-6 production [4].

A study comparing the immune response to *S. aureus* versus *S. epidermidis* concluded that Langerhans cells (LCs) and fibroblasts are essential to identifying MRSA and triggering inflammatory pathways. For example, LCs can differentiate *S. aureus* from *S. epidermidis* through their production of IL-1β. Similarly, fibroblasts produced more IL-8 when inoculated with MRSA compared to *S. epidermidis* [5]. This analysis demonstrates how, even though both *Staphylococcal* sp. have the potential to be skin commensals, tissue resident cells are able to differentiate between the pathogen MRSA and commensal bacteria such as *S. epidermidis*. Overall, *S. epidermidis* plays a lot of essential and beneficial roles for epithelial cells and contributes to the human innate immune system.

Various hypotheses exist regarding how *S. epidermidis* went from a benign bacterium to a serious threat. One hypothesis proposes that *S. epidermidis* produces more virulent mechanisms targeting immune system invasion rather than attacking the host [6]. This theory would explain how *S. epidermidis* became a commensal and a species that human hosts often do not respond to. It would also demonstrate why biofilm-associated infection is not often cleared by the human immune system. Furthermore, one study reports that *S. epidermidis* biofilm-grown strains elicit production of anti-inflammatory cytokines, which would explain why *S. epidermidis* is able to attenuate host responses. Another hypothesis suggests that *S. epidermidis* invasiveness is due to the acquisition of ica genes encoding production of PNAG/PIA and the insertion element IS256, with evidence depicting that ica-negative strains have a selective advantage over ica-positive strains [7]. This theory is also highly plausible given the recombinant nature of *S. epidermidis*, such as its ability to undergo horizontal gene transfer (HGT) and its predilection for coalescing into many lineages (See more Section 2.2.1).

### 2.2. Pathogenic S. epidermidis

In immunocompromised patients or patients with implanted mechanical devices, *S. epidermidis* inside the human body poses a serious threat. For example, *S. epidermidis* is the leading cause of infections in patients with prosthetic valves, cardiac devices, central lines, catheters, central nervous system (CNS) shunts, and even intravenous (IV) drug use [8]. In catheter-related infections, *S. epidermidis* as well as other coagulase-negative *Staphylococcus* (CoNS) can cause bloodstream infections. In more localized infections, patients may present with symptoms of inflammation such as erythema and purulence around the insertion site. In infections that progress systemically, patients can present with symptoms such as fever, hypotension, and other symptoms of sepsis.

Biofilms have gained much attention when it comes to *S. epidermidis*, as this species is one of the leading causes of infected prosthetic or native cardiac valves. Once biofilms are present, vegetations form, and infectious endocarditis can arise. Biofilm-producing strains are more adherent to surfaces such as cardiac valves and have a 10-fold higher intracellular survival rate in monocyte-derived macrophages. In response to biofilm-associated infections, the immune system elicits lower amounts of proinflammatory cytokines and Th1 response cytokines [9], making the patient more susceptible to bloodstream and subsequently, systemic infections. These patients can then present with fever, chills, malaise, night sweats, and dyspnea. These infections can also lead to sepsis, as *S. epidermidis* is the most frequent cause of nosocomial sepsis [6,8].

Furthermore, as infections become more understood over time, researchers have uncovered that *S. epidermidis* is an underrated cause of other diseases. This includes infectious mastitis in lactating women, which accounted for 200 out of 270 (74%) of *Staphylococcal* milk isolates [10]. Methicillin-resistant *S. epidermidis* has also been linked to infectious keratitis cases [11]. Moreover, overgrowth of *S. epidermidis* has been linked to several dermatoses, including seborrheic dermatitis, Netherton syndrome, and atopic dermatitis (AD) or eczema [12]. Strains causing AD may contain EcpA, a cysteine protease that disrupts the skin barrier [13]. In severe cases, *S. epidermidis* has the potential to cause necrotizing infections such as glandular and Fournier’s gangrene, specifically in diabetes mellitus (DM) patients [14,15].

Overall, it appears that *S. epidermidis* only appears pathogenic when exposed to compromised immune defenses or contamination events. Examples of this include its ability to cause disease within dysfunctional skin barriers, such as in AD and infectious mastitis, or immunocompromised hosts, such as DM patients developing Fournier’s gangrene. Additionally, immunocompromised patients have a higher risk and frequency of contamination events, such as requiring implanted devices, catheters, and IV lines, making them susceptible to biofilm-associated *S. epidermidis* infections.

#### 2.2.1. Virulence Factors

These bacteria can modulate their pathogenicity due to unique mechanisms. Much of the genetic variation in *S. epidermidis* is owed to its accumulation of resistance mechanisms, including adhesion, biofilm formation, genomic islands, phage elements, integrated plasmids, and quorum sensing.

Specific proteins that affect surface adhesion in *S. epidermidis*, such as the abundant surface protein, include AtlE. AtlE is a surface antigen that causes autolysis, which facilitates extracellular DNA release and therefore triggers cellular attachment. In a recent study, mupirocin was used to increase the expression of AtlE as well as CidA-LrgAB, a gene regulating the cell death system. By gene deletion of AtlE, biofilm production and eDNA release were eliminated [16].

Biofilm production is suggested to be caused by the icaD gene. Biofilm formation was studied on 44 strains of *S. epidermidis* isolated from female patients to check the expression of icaA, icaD, and aap genes. PCR was used, and four genotypic groups were divided into the IcaA+ icaD+/aap+ group (group A), the icaA+ icaD+/aap- group (group B), the icaA- icaD-/aap+ group (group C), and the icaA-icaD-/aap- group (group D). In total, 29 strains had bacterial biofilm formation (65.9%). There were 13 strains in group A, 7 strains in group B, 9 strains in group C, and group D did not form biofilms. The values of groups A, B, and C were higher than those of group D at 12–36 h, and group A was significantly higher than groups B and C (*p* < 0.05), but there was no difference between groups B and C (*p* > 0.05). Group A had the most abundant biofilm grown compared to the others. The results showed that icaA, icaD, and aap genes all help in biofilm formation, and the aap gene enhances the biofilm-forming ability of icaA and icaD genes [17].

#### 2.2.2. Resistance Mechanisms

In a meta-analysis of *S. epidermidis* genomes from five healthy patients, it was found that *S. epidermidis* isolates tend to coalesce into several founder lineages rather than a single colonizer isolate. Furthermore, comparison of these isolates revealed that the divergence was likely due to high rates of gene loss and HGT [18]. HGT involves the conjugation of plasmids from the donor to the recipient [19]. It is then replicated and transcribed into the double-stranded form. This process creates genetic diversity and can lead to an array of multidrug resistance [20].

A 2021 phage study demonstrated that the plasmids transferred between *S. epidermidis* clonal complexes share high overall identity to plasmids of *S. hominis*, *S. warneri*, *S. aureus*, and *Macrococcus caseolyticus* [21]. This study showed the first evidence of chromosomal island transfer and phage-mediated transduction in *S. epidermidis*. Plasmids play a key role in how *S. epidermidis* becomes pathogenic. Plasmids are small, circular extrachromosomal DNA that is found in bacterial species that are important in how the bacteria genetically adapt. They carry genes that offer resistance to antibiotics and can transcribe them to others through HGT alongside integrons, which help in the expression of external genes. Another critical component in the pathogenicity is the *Staphylococcal* cassette chromosome (*SCCmec*). This code for methicillin resistance hinders beta-lactam antibiotics, which can be transcribed to others, thus increasing pathogenicity. Genome islands are significant structures that aid in the evolution of *S. epidermidis.* These islands host genes that can increase the adaptability of the bacterial strains to different environments, including human hosts [22].

Overall, these mechanisms aid in the acquisition and circulation of genetic elements, thus enabling *S. epidermidis* to adapt from a commensal bacterium to a drug-resistant pathogenic species.

#### 2.2.3. Multidrug-Resistant Strains

While *S. epidermidis* has evolved with many resistance mechanisms, its multidrug-resistant strains pose a serious threat worldwide. Previous studies have found that *S. epidermidis* strains are most likely to be resistant to oxacillin (56%), gentamicin (44%), and erythromycin (41%). However, in healthy patients, only 10% of isolates demonstrated resistance, and only to penicillin [23]. However, in a more recent study comparing *S. epidermidis* isolates from healthy students, patients, and pasteurized milk, it was found that 32.3% of patient isolates showed some kind of resistance. The results showed that 89, 54, 60, and 14.2% of the strains were resistant to oxacillin, gentamycin, erythromycin, and fusidic acid, respectively. Strains from healthy students lacked the mecA gene, but still 32.3% of the student isolates had overall resistance across multiple drugs, which were amoxicillin/clavulanic acid, ampicillin, cefoxitin, cefotaxime, mupirocin, oxacillin, tetracyclines, and macrolides [24]. Not only are patients seeing an increased prevalence of antibiotic resistance, but healthy subjects are as well. In *S. epidermidis*, tetracycline resistance is mediated through active efflux or ribosomal protection mechanisms, while macrolide resistance arises from alterations of the ribosomal target or efflux-based processes. Although less common, resistance to last-line agents such as linezolid has been linked to modifications of the ribosomal target, raising concern about the expanding antimicrobial resistance reservoir [25].

Furthermore, *S. epidermidis* has been found to build more resistance to methicillin in nosocomial infections. A cross-sectional study found that methicillin resistance was observed in 75–90% of *S. epidermidis* isolates from nosocomial infections, which had a higher resistance rate than *S. aureus* isolates (40–60%). Additionally, 92.2% of *S. epidermidis* isolates carried the mecA gene [26]. Commonly, *S. epidermidis* isolates are reliably (>90%) susceptible to linezolid and vancomycin, but this is not true of all studies [27,28]. A 2022 retrospective from a 2007 to 2013 study identified three distinct multidrug-resistant *S. epidermidis* (MDRSE) species, and data from various isolates suggest that international dissemination of the three MDRSE has already occurred. Eighteen linezolid-resistant strains were found in Europe, and it is believed that the genetic determinants are acquisition of cfr, the G2576T substitution in domain V of 23S rRNA (equivalent to G2602T in *S. epidermidis*), and insertion of a glycine residue (71GR72 to 71GGR72) in ribosomal protein L4. Four vancomycin-resistant strains found in Germany also tested resistant to both daptomycin and linezolid, and D471E and I527M RpoB substitutions are believed to be the cause. One MDRSE lineage (BPH0662 ST2) is primarily dominant in Australia, but is in at least three more countries (Belgium, Denmark, and the United Kingdom), while another (ST23) is believed to be present in 7 countries (Australia, Austria, Belgium, Denmark, France, Germany, United States) [28].

## 3. Materials and Methods

A literature search was conducted on PubMed, Medline, CINAHL, and Google Scholar databases using algorithms with terms related to *Staphylococcus epidermidis*’ genomics. Titles and abstracts were screened by three independent reviewers to identify clinical studies, focused primarily on articles published between 2006 and 2025 that examined the evolution of *S. epidermidis* from a necessary symbiont to a pathogenic bacterium. Additional studies not captured in the initial database search from review articles were also assessed in this review. Clinical trials, interventional studies, retrospective chart reviews, systematic reviews, case series, and individual case reports were included in the literature review. Articles in languages other than English were included if an English translation was readily available. The findings from these publications were synthesized narratively, with a focus on the molecular mechanisms of *S. epidermidis* and the development and spread of multidrug-resistant strains.

No generative artificial intelligence (GenAI) was used in the writing of this manuscript.

## 4. Discussion

### 4.1. S. aureus Derived Genes

Studies suggest that the genomic diversity of *S. epidermidis* can be partially owed to other pathogenic bacteria such as *S. aureus*. Many studies have shown comparisons between the two species, and a large population functional genome analysis in 2015 revealed high levels of recombination in hospital isolates. Furthermore, 36 genes identified in the same study were shared by *S. aureus* and *S. epidermidis*, with only about 19% and 16% of genes unique to each species, respectively [29]. Approximately 1478 genes were universally present in all 181 *S. aureus* and 143 *S. epidermidis* isolates in this study, reflecting their shared evolutionary ancestry. While this core genome similarity demonstrates the close phylogenetic relationship between these species, horizontal gene transfer between *S. aureus* and *S. epidermidis* primarily involves mobile genetic elements and accessory genes rather than core genes.

More specifically, *S. aureus* and *S. epidermidis* both share similar virulence factors and are both capable of colonizing skin and implanted devices. In a study on breast prosthesis-related infections, researchers found *S. aureus* and *S. epidermidis* isolates from prosthesis demonstrated strong adhesion and biofilm production [30]. Another virulence factor that *S. epidermidis* produces is phenol-soluble modulin (PSM) peptides, a similar product derived from *S. aureus*. PSM peptides are believed to be cytotoxic to human keratinocytes and lead to skin inflammation. Since *S. aureus*, unlike the ubiquitous commensal *S. Epidermidis*, colonizes only a subset of individuals [31] and readily transitions to a pathogenic state, this overlap of PSM peptide production suggests that gene transfer of this inflammatory mechanism occurred from *S. aureus* to *S. epidermidis* [32]. PSM-mec, a PSM encoded within the mobile genetic element SCCmec, is produced by *S. epidermidis* and contributes to its virulence. PSM-mec has a cytolytic effect against neutrophils in vitro, and its presence has been associated with decreased bacterial clearance and higher mortality rate in a murine model of sepsis. In addition, certain members of the PSM family, such as PSMβ, are expressed at high levels during biofilm formation, contributing to the structuring and dispersal of biofilm [33].

Additionally, it is believed that gene transfer between the *Staphylococcus* species has contributed to the emergence of Methicillin-resistant *S. aureus* (MRSA) clones [34]. This likely contributed to *S. epidermidis* acquiring methicillin resistance through the *mecA* gene located in the *Staphylococcal* chromosome cassette mec (*SCCmec*) [35]. This resistance mechanism explains why both *S. aureus* and *S. epidermidis* are gaining traction as public health threats. Furthermore, the emergence of a pBSRC1 plasmid containing dual-biocide resistance is believed to originate from the interspecies DNA exchange between *S. aureus* and *S. epidermidis.* The pBSRC1 plasmid highlights the potential for mobile elements to promote persistence and dissemination of resistant *Staphylococcal* strains in environments where biocides are routinely used. Furthermore, the transfer of the arginine catabolic mobile element from *S. epidermidis* to *S. aureus* is thought to have expanded the ecological niche of *S. aureus* to the skin and potentiated cutaneous infections [36]. Several factors, including geography, disease, antibiotic use, and even household, seem to influence *Staphylococcal* diversity in both *S. aureus* and *S. epidermidis* [37].

In conclusion, *S. aureus* and *S. epidermidis* appear to share many genes, likely owing to both species’ predilection for high recombination rates. This also explains why both species have many immune evasion virulence factors and have many multidrug-resistant strains, giving them an advantage in causing high rates of infection in nosocomial settings.

#### 4.1.1. Biofilm Genomics

One of CoNS’ well-known virulence factors is its ability to form biofilms, which serve as a protective surface for *S. epidermidis* to avoid antibiotic exposure and the host’s immune defenses. As *S. epidermidis* has become more virulent, many genomic sequencing studies have been published to reveal its biofilm-associated genes. These genes include atlE, aae, ebpS, and fbe/sdrG, aap, and sesE. Specifically, the presence of the genes *aap* or *sesE* in *S. epidermidis* showed a significant association with increased biofilm formation [38]. Biofilm development involves three steps: initial adhesion, intercellular accumulation, and final detachment.

##### Initial Adhesion

During the initial adhesion stage, *S. epidermidis* binds to surfaces. Adhesion may occur on abiotic surfaces, such as bare plastics including catheters and prosthetic devices, or on biotic surfaces, including tissues or host extracellular matrix proteins that can be deposited on medical devices [34]. *S. epidermidis* undergoes protein-mediated, MSCRAMM-dependent adhesion, in which surface proteins encoded by specific adhesin genes bind defined host ligands [39]. Multiple MSCRAMM-encoding genes allow binding to different host matrix proteins, creating functional redundancy that protects against loss-of-function mutations in individual adhesins, as adhesive ability persists despite the inactivation of a single adhesin gene. This redundancy favors the *S. epidermidis* genome, preserving overlapping adhesion functions, allowing successful colonization across different tissues, device coatings, and host environments [34,39]. MSCRAMMs such as SdrG/Fbe, which binds fibrinogen, and Embp, which binds fibronectin, are sortase-anchored surface proteins whose primary function is adhesion [34,40]. Proteins such as *AtlE* and *Aae*, encoded by autolysin genes, are non-covalently surface-associated enzymes exhibiting secondary adhesive functions in addition to their roles in cell wall turnover. However, this process does not necessarily result in biofilm formation but establishes the genetic and structural foundation for persistent colonization and infection [41].

##### Intracellular Accumulation

Following the initial adhesion, which can be described as host binding to a surface, the intracellular accumulation phase involves bacterium-to-bacterium interactions mediated by intercellular adhesive properties. Further investigation of this mechanism identified polysaccharide intercellular adhesion (PIA) as a key component facilitating intercellular adhesion and structural integrity within *S. epidermidis* biofilms [42].

PIA is a high-molecular-weight polymer composed of β-1,6-linked N-acetylglucosamine residues. The synthesis of PIA is encoded by the intercellular adhesion (ica) gene locus. The proteins encoded by the two genes icaA and icaD function cooperatively to synthesize N-acetylglucosamine oligomers, with icaD serving to enhance the N-acetylglucosaminyltransferase activity of IcaA. In addition, a third gene, icaC, is required to produce longer polymer chains, creating PIA structures capable of mediating robust intercellular adhesion and forming the extracellular matrix characteristics of mature biofilms [43].

Following synthesis and export, the icaB-encoded deacetylase modifies PIA through partial deacetylation, introducing a positive charge that is essential for stable association of the polymer with the bacterial cell surface. This modification enhances PIA “stickiness,” promotes cell aggregation, and increases biofilm stability through electrostatic interactions [44].

Expression of the icaADBC operon is tightly regulated by icaR, which encodes a transcriptional repressor that binds upstream of icaA, leading to suppression of transcription of the biosynthetic genes. Environmental cues, including stress conditions encountered during host colonization, modulate PIA production largely through the regulation of icaR. Deletion or inactivation of icaR results in overproduction of PIA, emphasizing its key role as a negative regulator of intercellular adhesion and biofilm formation [45]. In addition to icaR-mediated repression, PIA production is influenced by global regulatory factors such as the alternative sigma factor σ^B and the *Staphylococcal* accessory regulator SarA. The sigma factor σ^B represses the icaADBC operon indirectly through upregulation of icaR, whereas SarA directly enhances icaADBC transcription and PIA production, demonstrating the presence of independent regulatory pathways that fine-tune biofilm synthesis in response to stress [46].

Functionally, PIA serves not only as a structural component of the biofilm matrix but also as an important mediator of immune evasion. The partially deacetylated and positively charged poly-β-1,6-N-acetylglucosamine (PNAG) polymer promotes bacterial aggregation during the accumulation phase of biofilm development. PIA-rich biofilms demonstrate increased resistance to host immune defenses, shielding bacteria from phagocytosis and antimicrobial peptides. Antibodies directed against PIA preferentially recognize secreted polymers rather than the surface-associated polymer, rendering natural immune responses less effective [47].

Although PIA-mediated accumulation represents the dominant mechanism of biofilm development, many isolates have been shown to lack the icaADBC operon. The presence of the icaADBC operon is heterogeneously distributed among S. epidermidis isolates and does not uniformly predict biofilm formation. In a clinical isolate collection, approximately 68% of strains carried the icaADBC operon, while only around 46% exhibited a biofilm-forming phenotype, demonstrating a clear discordance between genotype and phenotype. The loss of the ica locus does not necessarily result in complete abolishment of accumulation but instead indicates the existence of alternative pathways. Certain *S. epidermidis* strains are capable of PIA-independent accumulation through the use of the accumulation-associated protein (Aap). This functional redundancy and strain variability support the presence of parallel accumulation strategies that enhance bacterial survival under diverse environmental conditions. Successful accumulation ultimately leads to multilayered biofilm development, which contributes to persistent and resistant infections [9,48].

##### Detachment

**Figure 1:**
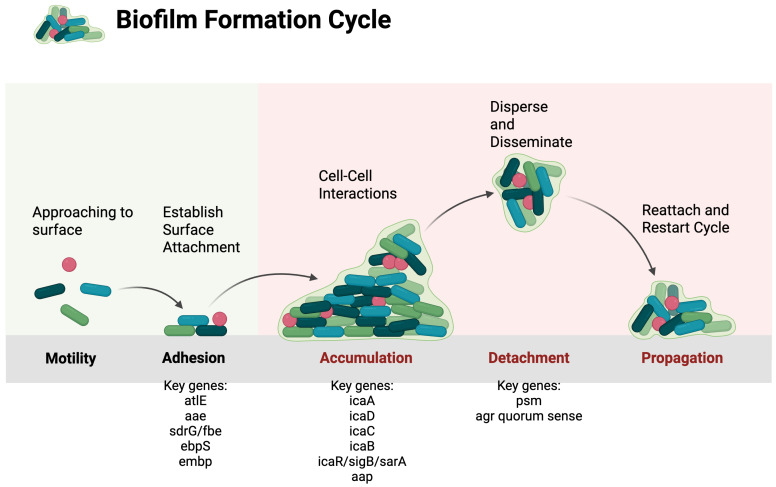
Biofilm formation cycle of *S. epidermidis*. Schematic overview of the biofilm life cycle, including initial surface approach and adhesion, intercellular accumulation, detachment (dispersal), and propagation through reattachment at secondary sites. Created in BioRender. Dela Cruz, W. (2026) https://BioRender.com/leddmbt (10 January 2026).

Finally, detachment, or biofilm dispersal, represents the last phase in the biofilm life cycle, during which cells actively escape the mature biofilm matrix and disperse into the surrounding environment. This process is important for bacterial survival and transmission. Detachment allows translocation of cells to new locations, followed by reattachment to a substrate at secondary sites. Dispersal can occur through either active or passive mechanisms. Active detachment is initiated by the bacteria, whereas passive detachment is mediated by external forces such as shear stress or mechanical abrasion [49]. Mechanistically, biofilm detachment is driven by regulated weakening of the biofilm matrix through production of PSMs, which disrupt intercellular interactions and promote the release of bacterial cells, as seen in Figure 1 [16].

In *S. epidermidis*, PSMs are surfactant-like peptides that contribute to biofilm disassembly and are regulated by the agr quorum-sensing system. The timing and extent of disassembly are locally regulated, such that favorable conditions may promote continuous release of small numbers of cells or sporadic large-scale detachment following prolonged periods of growth. Under nutrient-rich conditions, glucose represses the agr system through acidification of the local environment, resulting from the excretion of acidic metabolites. When glucose becomes depleted, agr activity is restored, leading to the production of matrix-degrading enzymes and resumption of biofilm disassembly. Thus, changes in nutrient availability serve as an important trigger for dispersal [50].

Dispersed cells tend to be more susceptible to antimicrobial agents and host immune responses. However, cells released during dispersal also exhibit a distinct phenotypic state characterized by enhanced virulence and altered susceptibility to antimicrobial agents, contributing to disease progression and treatment failure [51]. Biofilm detachment further facilitates the dissemination of bacteria into the bloodstream, which can lead to catheter-related bloodstream infections as disseminated bacteria seed new sites of infection. Collectively, these observations highlight detachment as both a driver of pathogenesis and an opportunity for therapeutic interventions aimed at manipulating dispersal to enhance infection control [52].

### 4.2. Antibiotic Resistance Genomics

Recent literature confirmed the usage of a major facilitator superfamily (MFS) efflux pump by the *Staphylococci* genus, called the nms gene. Strains of *S. aureus* and *S. epidermidis* were revealed to contain this gene, including *S. epidermidis* ST570/ST1166 strains from global isolates. The *nms* gene functions as a 12-transmembrane segment MFS efflux pump that effectively extrudes various antimicrobial agents, conferring multidrug resistance through active efflux mechanisms. Phylogenetic analysis revealed that nms-positive strains form a distinct clade, with the gene likely originating from plasmids before integrating into chromosomes, as evidenced by significant plasmid-related mobile genetic elements and circular intermediates showing high homology with *S. aureus* plasmids. The nms gene is embedded within a genomic island that is believed to be originally derived from *S. epidermidis* [53,54].

Beyond the nms gene, the antibiotic resistance genomics of *S. epidermidis* reveals a complex landscape shaped by mobile genetic elements and HGT. Comprehensive genomic analyses have identified core antibiotic resistance genes, including dfrC (dihydrofolate reductase) and norA (multidrug efflux pump), present across *S. epidermidis* genomes, with β-lactam resistance genes showing particularly high prevalence [55].

The staphylococcal cassette chromosome mec (*SCCmec*) represents the primary mobile genetic element conferring methicillin resistance through the *mecA* gene, with *S. epidermidis* harboring remarkable *SCCmec* diversity, including types I through VIII and numerous untypeable variants [56,57]. Approximately 70–80% of *S. epidermidis* isolates carry *mecA*, and many strains harbor multiple ccr gene complex copies, creating a vast reservoir for *SCCmec* evolution and dissemination [58]. The insertion sequence IS256 shows strict association with multidrug resistance, particularly gentamicin resistance, as aminoglycoside resistance genes frequently co-exist with IS256 within *Staphylococcal* transposons [59]. HGT between *S. epidermidis* and *S. aureus* occurs through multiple mechanisms, including phage-mediated transfer governed by wall teichoic acid (WTA) structure compatibility and conjugative plasmid-mediated *SCCmec* transfer [60,61,62].

Hospital-adapted *S. epidermidis* lineages (ST2 and ST23) have acquired *S. aureus*-type ribitolphosphate WTA through the accessory tarIJLM element, enabling efficient DNA exchange via siphovirus bacteriophages and facilitating interspecies transfer of resistance determinants [61]. Documented cases confirm in vivo *SCCmec* transfer from *S. epidermidis* to methicillin-susceptible *S. aureus* during antibiotic therapy, with transferred elements showing >99% sequence identity [28]. Prolonged nosocomial exposure has driven dramatic genomic remodeling in *S. epidermidis*, characterized by phage loss, *SCCmec* and insertion sequence acquisition, and accumulation of multiple resistance genes [63].

Modern hospital-adapted strains exhibit resistance to rifampicin through specific *rpoB* mutations (D471E and I527M) that not only confer rifampicin resistance but also reduce susceptibility to vancomycin and teicoplanin, creating near pan-drug-resistant phenotypes [64]. The global spread of three multidrug-resistant lineages (two ST2 and one ST23) demonstrates the successful adaptation of *S. epidermidis* to the hospital environment, with these lineages now distributed across 96 institutions in 24 countries [64]. Additional resistance mechanisms include chloramphenicol acetyltransferase genes (*cat*) acquired from *Enterococcus faecium* plasmids via IS6 family transposases, demonstrating horizontal gene transfer between Gram-positive pathogens [55]. The lack of CRISPR sequences and specific restriction-modification profiles in *S. epidermidis* may facilitate genetic exchange of *SCCmec* elements among different *Staphylococcal* species [53]. This genomic plasticity, combined with the organism’s role as a resistance gene reservoir, positions *S. epidermidis* as a critical contributor to the evolution of antibiotic resistance in clinically significant *Staphylococci*.

### 4.3. Combating Multi-Drug Resistance

#### 4.3.1. Antibiotic Combination Therapies

Combating MDRSE infections requires a multifaceted approach that addresses both planktonic bacteria and biofilm-associated organisms. Vancomycin remains the cornerstone therapy for methicillin-resistant *S. epidermidis* (MRSE) infections, though emerging alternatives demonstrate comparable or superior efficacy in specific clinical contexts [65,66]. Daptomycin has shown promise, demonstrating superior efficacy compared to vancomycin in experimental models of foreign-body and systemic MRSE infections, with significant reductions in bacterial loads in both liver tissue and catheter-associated biofilms [66]. Notably, recent investigations have revealed cryptic susceptibility to penicillin/β-lactamase inhibitor combinations such as amoxicillin/clavulanic acid in hospital-adapted MRSE lineages despite *mecA* carriage, offering a potential therapeutic avenue that warrants further clinical investigation [67]. Combination therapies have demonstrated enhanced bactericidal activity, with rifampin augmentation significantly improving outcomes when combined with vancomycin, linezolid, or daptomycin in experimental osteitis models, achieving complete sterilization in most cases after two weeks of treatment [57,68,69]. Additionally, β-lactam antibiotics such as nafcillin and ceftaroline can augment daptomycin killing of MRSE through increased binding to bacterial surfaces, a mechanism that also enhances susceptibility to host antimicrobial peptides like cathelicidin LL-37 [65].

#### 4.3.2. Antimicrobial Peptides

Antimicrobial peptides (AMPs) represent a promising alternative therapeutic class with distinct mechanisms of action that circumvent traditional resistance pathways. The Cec4-derived peptide C9 exhibits rapid bactericidal activity against MRSE with minimal inhibitory concentrations of 8 μg/mL, demonstrating both antibiofilm properties and the ability to prevent high-level resistance development through multiple nonspecific cellular targets, including membrane disruption, DNA binding, and oxidative stress induction [70,71,72]. AMPs offer broad-spectrum activity against multidrug-resistant *Staphylococci* while maintaining low propensity for resistance development due to their membrane-targeting mechanisms and immunomodulatory properties [73]. Synthetic molecular evolution has enabled optimization of AMPs to overcome traditional limitations such as proteolytic susceptibility, host cell toxicity, and loss of activity in protein-rich environments, yielding candidates with potent sterilizing activity against ESKAPE (*Enterococcus faecium*, *Staphylococcus aureus*, *Klebsiella pneumoniae*, *Acinetobacter baumannii*, *Pseudomonas aeruginosa*, and *Enterobacter species*) pathogens and biofilm-forming bacteria, even in complex wound environments [74]. Despite their therapeutic potential, AMPs face challenges, including economic and regulatory obstacles that have limited clinical translation, though several candidates have advanced to clinical trials for staphylococcal infections [75].

#### 4.3.3. Chlorhexidine Antiseptic

Biofilm disruption strategies are critical for eradicating device-associated MRSE infections, as standard systemic antibiotic concentrations fail to penetrate and eradicate established biofilms. Chlorhexidine digluconate (CHG), a widely used antiseptic in clinical practice, was used in antimicrobial susceptibility assays against *S. epidermidis* and found that CHG had antimicrobial effects in both suspension and biofilm (MIC 2–8 mg/L) [76]. They suggested essential oil adjuvants to CHG, including eucalyptus oil. Another more recent study used chlorhexidine irrigation at concentrations of 0.05% and 0.1%, which effectively eradicated *S. epidermidis* from biofilm in vitro at clinically relevant exposure times, demonstrating superior efficacy compared to povidone-iodine, sodium hypochlorite, and triple antibiotic solutions [77].

However, a novel qacA allele has been introduced and has already indicated reduced susceptibility in *S. epidermidis*. This qacA encodes a 514-amino-acid, 14-transmembrane-segment pump with the capacity to efflux CHG [78], so CHG use alone is not recommended in the case of the widespread qacA allele. Reduced susceptibility to antiseptics in *S. epidermidis* is more accurately described as antiseptic tolerance rather than complete resistance. This tolerance is primarily mediated by efflux mechanisms such as qacA, which increase CHG minimum inhibitory concentrations and enable bacterial survival under sublethal exposure conditions. Clinically, the widespread use of CHG for skin antisepsis, device irrigation, and infection prevention may select for tolerant strains, facilitating biofilm persistence in healthcare settings. Therefore, ongoing surveillance of antiseptic susceptibility is essential to limit the emergence and dissemination of tolerant, multidrug-resistant *S. epidermidis* strains [77,78].

#### 4.3.4. Rifampin-Based Therapies

Supratherapeutic antibiotic concentrations achievable through local delivery methods show promise, with daptomycin at 500 μg/mL alone capable of eradicating high-biofilm-forming isolates, while levofloxacin combined with rifampin at supratherapeutic doses achieved biofilm eradication within 48 h [79].

Adjunctive agents such as N-acetylcysteine combined with rifampin produce significant 4-log reductions in biofilm bacterial loads through complementary mucolytic and antimicrobial mechanisms [80,81,82]. Novel surface modification strategies employing small molecules that interfere with bacterial nucleotide signaling (c-di-AMP, c-di-GMP, cAMP) demonstrate dual benefits of inhibiting biofilm formation and increasing antibiotic efficacy, with ciprofloxacin effectiveness improving from 94.8% to >99.9% on modified surfaces [83]. Targeting persistent cell subpopulations within biofilms through extended exposure to antibiotics at biofilm minimum bactericidal concentrations represents an additional strategy for achieving complete biofilm eradication, particularly relevant for antibiotic lock techniques and aerosolized antibiotic delivery.

#### 4.3.5. Phage Therapy

In recent years, phage therapy has gained momentum as antibiotic resistance has become a substantial threat. So far, phage therapy has been promising in the fight against *S. aureus*, specifically against MRSA [84,85]. However, there is limited data on using phages against *S. epidermidis*. Phage therapy against *S. epidermidis* has honed in on its ability to form biofilms on catheters. However, it has only been found to be effective depending on biofilm characteristics like age, strength, and structure, among others [86]. One phage, CUB-EPI_14, was able to eliminate (no detection of viable cells) the biofilm of SE16 after 10 h co-incubation [87], while another phage (*vB_SepM_BE04*) was capable of killing staphylococcal cells within biofilms specifically formed on polyurethane catheters [88]. The only two recent reports of successful phage therapy in humans were for the treatment of a life-threatening periprosthetic joint infection with MDRSE [89] and treatment of a left ventricular assist device infection [90]. Due to limited data on humans, it is unclear how sufficient phage therapy can be against MDRSE alone, but phages may be used alongside antibiotics in the near future, especially as researchers learn to direct phage evolution [86].

Overall, there is significant room for improvement regarding *Staphylococci* therapeutics, and there is an abundance of literature focused on combating *S. epidermidis* and *S. aureus* biofilms. More research needs to be done on nonconventional therapies to reduce the likelihood of *S. epidermidis* gaining resistance to more antibiotic therapies. A comprehensive summary of alternative therapeutic strategies evaluated against multidrug-resistant *S. epidermidis* is provided in Table S1 (Supplementary Materials).

## 5. Conclusions

*Staphylococcus epidermidis* plays a crucial part in the human skin microbiome and has been regarded as a beneficial symbiotic bacterium due to its role in innate immune modulation. However, under certain conditions, such as in clinical settings, medical device implants, and immunocompromised individuals, this commensal bacterium becomes an opportunistic pathogen. This shift is mainly due to biofilm formation and antibiotic resistance, enabling it to evade the hosts’ defenses and evade a plethora of antibiotics. Many genomic studies have uncovered the many genes that *S. epidermidis* has acquired, along with locating its pan-resistant lineages. Now, it is important to utilize this abundance of knowledge, but against *S. epidermidis* to prevent its persistent evolution and the evolution of species that benefit from it. Worldwide surveillance, novel antibiotic combinations, and research focused on alternative therapeutics are monumental in combating this resilient and troublesome species from global takeover.

## Data Availability

No new data were created in the production of this literature review. The data presented in this study were derived from the following resources available in the public domain(s) of: https://pubmed.ncbi.nlm.nih.gov/ (10 January 2026).

## Supplementary Materials

The following supporting information can be downloaded at: https://www.mdpi.com/article/10.3390/biom16020334/s1. Table S1: Alternative therapies for combating multidrug resistance. This table summarizes the findings on methods of combating the multidrug resistance of *S. epidermidis*, illustrating the limited alternative therapies and the concentrations in which they have proved maximal efficacy. References [67,68,69,70,72,76,77,81,82,86,88,89,90,91,92] are cited in Supplementary Materials.

## Abbreviations

The following abbreviations are used in this manuscript:

CoNS	Coagulase-negative Staphylococci
MRSA	Methicillin-resistant Staph aureus
MDRSE	Multidrug-resistant Staph epidermidis
HGT	Horizontal gene transfer
