# Supplementary material for: The Evolution of Symbiosis in Staphylococcus epidermidis: From a Protective Mutualist to a Parasitic Pathogen
Source: Biomolecules. 2026 Feb 23;16(2):334. doi: 10.3390/biom16020334 (PMC12938455; doi:10.3390/biom16020334)
Supplement: Supplementary file 1 [file biomolecules-16-00334-s001.zip › biomolecules-4134791-Supplementary Materials.pdf]

Supplementary Materials:

Table S1. Alternative therapies for combating multidrug resistance. This table summarizes the findings on methods of combatting the multidrug resistance of *S. epidermidis*, illustrating the limited alternative therapies and the concentrations in which they have proved maximal efficacy.

Method	*Mechanism of Action (MoA)*	*Evidence*
**Amoxicillin/Clavulanic acid**	Biofilms seemed to decline with increasing concentrations of amoxicillin/clavulanic acid. The combination of amoxicillin and clavulanic acid prevents amoxicillin from being degraded by beta-lactamases.	*Ba et al.* results support that penicillin/β-lactamase inhibitor combinations could be a promising therapeutic candidate for short-term treatment of non-biofilm-related MRSE infections. Amoxicillin/clavulanic acid also had a partial effect on MRSE biofilms and thus might be useful in combination with rifampicin or other antibiotics [67].
**Rifampin/rifampicin therapies**	Adding rifampin seems to decrease the Minimum Biofilm Eradication Concentration (MBEC). Previous studies have found that rifampin has enhanced activity against bacteria in biofilms.	*Stavrakis et al.* found that the addition of rifampin to low-dose cefazolin and vancomycin was highly effective in further reducing the CFU harvested from the implants [68].<br>*Jorgensen et al.* found that treatment with linezolid and daptomycin was comparable to vancomycin, in combination with rifampicin. Additionally, combinations containing two non-rifampicin antibiotics were not more active than single drugs [69].<br>*Bazrgari et al.* used liposomal rifampin, and reported it was the most effective formulation against *S. epidermidis* [68].
**Antimicrobial peptides (AMPs)**	The MoA of these peptides may vary and it is believed that AMPs can target bacterial cell membranes by lipid bilayer disruption, interfere with the nucleic acids of the targeted cell or act on the specific YycG/YycF system.<br><br>The AMP C9 in particular can exert antibacterial effects by destroying the cell membrane structure, changing the cell membrane permeability, depolarization level, and cell membrane fluidity, and triggering ROS accumulation.	*Agrawal et al.* concluded the combination of natural and synthetic peptides with antibiotics has reduced its MIC and lowered the chances for the development of resistant strains [72].<br><br>*Mao et al.* reported that the peptide C9 has rapid bactericidal and antibiofilm activity and the potential to avoid the development of resistance. In addition, it has therapeutic potential against *S. epidermidis* infection *in vivo* [70].
**Chlorhexidine**	Potent membrane-active agent against bacteria and inhibits outgrowth, but not germination, of bacterial spores, although it is not sporicidal [91].	*Karpanen et al.* demonstrated CHG exhibited antimicrobial activity against *S. epidermidis* in both suspension and biofilm (MIC 2-8 mg/L) [76].<br>*Schmidt et al.* found that chlorhexidine 0.05% and 0.1%, povidone-iodine 10%, and povidone-iodine 3.5% at 10 minutes were effective at eradicating *S. epidermidis* from biofilm [77].
**N-acetylcysteine (4mg/mL) combined with rifampicin (10mg/L)**	NAC has a mucolytic effect on biofilms.	*Leite et al.* expressed significantly reduced (4 log10) the number of biofilm cells and high concentrations of NAC is bactericidal to *S. epidermidis* in both planktonic and biofilm states [81].

		Eroshenko et al. found three strains of coagulase-negative *Staphylococci* (CNS 19, CNS 33, CNS 34), and *S. aureus* ATCC 25923, were reduced in the presence of ≥ MIC of NAC. However, NAC at a sub-MIC in some cases even enhance biofilm formation [82].
**Phage adjuvants**	Phages may be able to selectively infect and lyse bacterial cells.	*Valente et al.* highlighted that one of the phages (vB_SepM_BE04) was capable of killing *Staphylococcal* cells within biofilms formed on polyurethane catheters [88].<br>*Cammuso et al.* developed a novel phage protocol for MDRSE in a joint infection with twice daily, intraarticular and IV phage delivery for 14 days. It resulted in healing of the wound within 1 month after treatment [89].<br>*Pitton et al. describes* vB_SepS_BE22 reduced bacterial loads in exponential and stationary phase cultures. When combined with rifampicin, it reduced biofilms on drivelines as well [90].<br>*Duarte et al.* study support the potential of the virulent phage AICAT, having a wide host range against *S. epidermidis* but its efficacy is still limited, even when combined with vancomycin, making it necessary to explore additional combinations with other phages or antimicrobials [92].<br>*Valdivia et al.* utilized staphylophage under biofilm and planktonic conditions and was able to evolve the lytic phage *vB_Sep_Steph1* to improve antibacterial efficacy and replicative fitness [86].

67. Ba, X.; Raisen, C. L.; Restif, O.; Cavaco, L. M.; Vingsbo Lundberg, C.; Lee, J. Y. H.; Howden, B. P.; Bartels, M. D.; Strommenger, B.; Harrison, E. M.; et al. Cryptic susceptibility to penicillin/β-lactamase inhibitor combinations in emerging multidrug-resistant, hospital-adapted Staphylococcus epidermidis lineages. *Nat. Commun.* **2023**, *14*, 6479. https://doi.org/10.1038/s41467-023-42245-y.
68. Stavrakis, A. I.; Niska, J. A.; Shahbazian, J. H.; Loftin, A. H.; Ramos, R. I.; Billi, F.; Francis, K. P.; Otto, M.; Bernthal, N. M.; Uslan, D. Z.; et al. Combination prophylactic therapy with rifampin increases efficacy against an experimental Staphylococcus epidermidis subcutaneous implant-related infection. *Antimicrob. Agents Chemother.* **2014**, *58*, 2377–2386. https://doi.org/10.1128/AAC.01943-13.
69. Jørgensen, N. P.; Skovdal, S. M.; Meyer, R. L.; Dagnæs-Hansen, F.; Fuursted, K.; Petersen, E. Rifampicin-containing combinations are superior to combinations of vancomycin, linezolid and daptomycin against Staphylococcus aureus biofilm infection in vivo and in vitro. *Pathog. Dis.* **2016**, *74*, ftw019. https://doi.org/10.1093/femspd/ftw019.
70. Mao, C.; Wang, Y.; Yang, Y.; Li, L.; Yuan, K.; Cao, H.; Qiu, Z.; Guo, G.; Wu, J.; Peng, J. Cec4-Derived Peptide Inhibits Planktonic and Biofilm-Associated Methicillin Resistant Staphylococcus epidermidis. *Microbiol. Spectr.* **2022**, *10*, e02409-22. https://doi.org/10.1128/spectrum.02409-22.
72. Agarwal, S.; Sharma, G.; Dang, S.; Gupta, S.; Gabrani, R. Antimicrobial Peptides as Anti-Infectives against Staphylococcus epidermidis. *Med. Princ. Pract.* **2016**, *25*, 301–308. https://doi.org/10.1159/000443479.
76. Karpanen, T. J.; Worthington, T.; Hendry, E. R.; Conway, B. R.; Lambert, P. A. Antimicrobial efficacy of chlorhexidine digluconate alone and in combination with eucalyptus oil, tea tree oil and thymol against

planktonic and biofilm cultures of Staphylococcus epidermidis. *J. Antimicrob. Chemother.* **2008**, *62*, 1031–1036. https://doi.org/10.1093/jac/dkn325.

77. Schmidt, K.; Estes, C.; McLaren, A.; Spangehl, M. J. Chlorhexidine Antiseptic Irrigation Eradicates Staphylococcus epidermidis From Biofilm: An In Vitro Study. *Clin. Orthop. Relat. Res.* **2018**, *476*, 648–653. https://doi.org/10.1007/s11999.0000000000000052.
81. Leite, B.; Gomes, F.; Teixeira, P.; Souza, C.; Pizzolitto, E.; Oliveira, R. Staphylococcus epidermidis biofilms control by N-acetylcysteine and rifampicin. *Am. J. Ther.* **2013**, *20*, 322–328. https://doi.org/10.1097/MJT.0b013e318209e17b.
82. Eroshenko, D.; Polyudova, T.; Korobov, V. N-acetylcysteine inhibits growth, adhesion and biofilm formation of Gram-positive skin pathogens. *Microb. Pathog.* **2017**, *105*, 145–152. https://doi.org/10.1016/j.micpath.2017.02.030.
86. Valdivia, C.; Domingo-Calap, P. Directed evolution of a staphylophage under biofilm and planktonic conditions. *Npj Biofilms Microbiomes* **2026**, *12*, 27. https://doi.org/10.1038/s41522-025-00893-6.
88. Valente, L. G.; Pitton, M.; Fürholz, M.; Oberhaensli, S.; Bruggmann, R.; Leib, S. L.; Jakob, S. M.; Resch, G.; Que, Y. A.; Cameron, D. R. Isolation and characterization of bacteriophages from the human skin microbiome that infect Staphylococcus epidermidis. *Microbes* **2021**, *2*, xtab003. https://doi.org/10.1093/femsmc/xtab003.
89. Cammuso, M. T.; Cook, B. W. M.; Cameron, D. W.; Ryan, S.; Tamayo, M.; Peters, M. J.; Arnaud, T.; Lau, S.; Almblad, H.; Fournier, N., et al. First Use of Phage Therapy in Canada for the Treatment of a Life-Threatening, Multidrug-Resistant Staphylococcus epidermidis Periprosthetic Joint Infection: An N-of-1 Trial. *Viruses* **2025**, *17*, 1118. https://doi.org/10.3390/v17081118.
90. Pitton, M.; Valente, L. G.; Oberhaensli, S.; Gözel, B.; Jakob, S. M.; Sendi, P.; Fürholz, M.; Cameron, D. R.; Que, Y. A. Targeting Chronic Biofilm Infections With Patient-derived Phages: An In Vitro and Ex Vivo Proof-of-concept Study in Patients With Left Ventricular Assist Devices. *Open Forum Infect. Dis.* **2025**, *12*, ofaf158. https://doi.org/10.1093/ofid/ofaf158.
91. Russell, A. D. Chlorhexidine: antibacterial action and bacterial resistance. *Infection* **1986**, *14*, 212–215. https://doi.org/10.1007/BF01644264.
92. Duarte, A. C.; Fernandez, L.; Rodriguez, A.; Garcia, P. A new bacteriophage infecting Staphylococcus epidermidis with potential for removing biofilms by combination with chimeric lysin CHAPSH3b and vancomycin. *ASM J. Msphere* **2025**, *10*, e01014-24. https://doi.org/10.1128/msphere.01014-24.
